# QTL detection and putative candidate gene prediction for leaf rolling under moisture stress condition in wheat

**DOI:** 10.1038/s41598-020-75703-4

**Published:** 2020-10-29

**Authors:** Aakriti Verma, M. Niranjana, S. K. Jha, Niharika Mallick, Priyanka Agarwal, Vinod

**Affiliations:** Division of Genetics, ICAR-Indian Agricultural Research Institute, New Delhi, 110012 India

**Keywords:** Genetics, Molecular biology, Plant sciences

## Abstract

Leaf rolling is an important mechanism to mitigate the effects of moisture stress in several plant species. In the present study, a set of 92 wheat recombinant inbred lines derived from the cross between NI5439 × HD2012 were used to identify QTLs associated with leaf rolling under moisture stress condition. Linkage map was constructed using Axiom 35 K Breeder’s SNP Array and microsatellite (SSR) markers. A linkage map with 3661 markers comprising 3589 SNP and 72 SSR markers spanning 22,275.01 cM in length across 21 wheat chromosomes was constructed. QTL analysis for leaf rolling trait under moisture stress condition revealed 12 QTLs on chromosomes 1B, 2A, 2B, 2D, 3A, 4A, 4B, 5D, and 6B. A stable QTL *Qlr.nhv-5D.2* was identified on 5D chromosome flanked by SNP marker interval *AX-94892575*–*AX-95124447* (5D:338665301–5D:410952987). Genetic and physical map integration in the confidence intervals of *Qlr.nhv-5D.2* revealed 14 putative candidate genes for drought tolerance which was narrowed down to six genes based on in-silico analysis. Comparative study of leaf rolling genes in rice viz., *NRL1*,* OsZHD1*,* Roc5*, and *OsHB3* on wheat genome revealed five genes on chromosome 5D. Out of the identified genes, *TraesCS5D02G253100* falls exactly in the QTL *Qlr.nhv-5D.2* interval and showed 96.9% identity with *OsZHD1*. Two genes similar to *OsHB3 *viz*. TraesCS5D02G052300* and *TraesCS5D02G385300* exhibiting 85.6% and 91.8% identity; one gene *TraesCS5D02G320600* having 83.9% identity with *Roc5* gene; and one gene *TraesCS5D02G102600* showing 100% identity with *NRL1* gene were also identified, however, these genes are located outside *Qlr.nhv-5D.2* interval. Hence, *TraesCS5D02G253100* could be the best potential candidate gene for leaf rolling and can be utilized for improving drought tolerance in wheat.

## Introduction

Bread wheat (*Triticum aestivum* L.) is one of the world's major staple food crops accounting for 20% of calories consumed globally^1^. Global wheat production amounts to 771 million tonnes (mt) of grains from an area of 218 million hectares (mha)^2^. Drought is among one of the major stress factors affecting wheat yields, especially in rain fed agriculture. As much as 21 percent reduction in wheat yield has been observed at approximately 40% water reduction^3^. Almost 50% of cultivation in the developing countries occurs in rain fed system^4^. Besides, the ongoing climate change crisis is making the problem far worse with infrequent and erratic precipitation leading to the expansion of drought prone areas. As much as 50% productivity reduction in wheat is observed in many countries in drought years leading to significant per capita food production decline^5–7^. Breeding drought tolerant cultivars is the best strategy for mitigating the effects of water deficit conditions. Drought tolerance is a complex trait involving several morphological, agronomical, physiological, and biochemical components. Drought stress is the result of an imbalance between evapo-transpiration flux and water intake from soil^8^. Moisture stress conditions result in physiological dehydration at the cellular level^9^. Response of plants to moisture stress at cellular level is complex and involves several factors including signaling molecules, transcription factors, hormones, and secondary metabolites^7^. Hence, estimation of drought tolerance is a difficult task. From the breeders’ point of view, drought tolerance is the ability of plants to survive moisture stress conditions with minimum reduction in yield. Response of plants to drought stress is broadly classified into two categories: avoidance and tolerance. Avoidance mechanisms include morphological and physiological adjustments such as reduced stomatal number and conductance, increased leaf thickness and decreased leaf area, increased root system and leaf rolling to reduce evapo-transpiration and thus enabling plants to escape moisture stress^7, 10, 11^. Tolerance mechanisms, on the other hand, maintain cellular turgor pressure by biochemical modifications through osmotic adjustments^12^. Leaf rolling is an escape mechanism evolved by plants to increase water use efficiency under moisture deficit conditions. In wheat, leaf lamina rolls transversally along the mid-rib to form a cylinder under severe moisture stress and/or high temperature and unrolls when stress is relieved^13^. This enables the plant to conserve water by decreasing transpiration and reducing leaf temperature. Wheat genotypes showing this trait exhibit better stomatal regulation, higher water use efficiency (WUE), and photosynthetic efficiency under stress conditions^14^.

Leaf rolling is a complex quantitative trait that is controlled by multiple genes following non-Mendelian inheritance^15^. Leaf rolling is a typical response related to mechanism of reducing moisture stress in field crops which is well studied in rice^16, 17^ and maize^15, 18^. Five mutants in maize^19–22^ and 17 mutants in rice with leaf rolling traits^22^ have been characterized. Additionally, more than 70 genes/QTLs associated with leaf rolling have been mapped in rice^23^. In contrast, studies regarding leaf rolling and identification of associated genomic regions or genes in wheat are scarcely reported. A study on RIL (recombinant inbred lines) population developed by crossing durum wheat (*T. turgidum* ssp. *durum*) with wild emmer wheat (*T. turgidum* ssp. *dicoccoides*) identified 11 significant QTLs associated with flag leaf rolling^24^. In another related species *Secale cereale* (Rye), four stable QTLs associated with leaf rolling were reported on chromosomes 3R, 5R, and 7R^25^ utilizing DArT markers. Wheat is a polyploid species and identifying genes with minor effects like those affecting drought tolerance is a daunting task. Quantitative trait loci (QTL) mapping using biparental populations is one of the commonly used approaches to analyze complex traits to identify genomic regions associated with complex traits such as drought tolerance. Successful QTL mapping requires high density of markers for precise QTL detection and accurate identification of candidate genes for complex traits. Earlier, SSR and other DNA based markers have been utilized for QTL mapping. However, with the advent of single nucleotide polymorphism (SNPs) genotyping platforms and availability of whole genome sequence of bread wheat^26^ it has become possible to develop high density molecular maps with greater precision.

In the present study leaf rolling trait was investigated in a mapping population comprising of RILs derived from the cross NI5439 x HD2012. Array based SNP markers along with SSR markers were used for generating a high-density linkage map. Polymorphic SNP and SSR markers were utilized for QTL mapping of leaf rolling. The objectives of this study were: (1) to create a genetic linkage map utilizing the SNP markers derived using 35 K SNP Array; (2) to identify QTLs controlling leaf rolling trait; (3) to infer potential candidate gene(s) responsible for leaf rolling under moisture stress and (4) comparison of leaf rolling genes predicted in wheat with cloned leaf rolling genes in rice.

## Results

### Genetic linkage mapping

**Figure 1:**
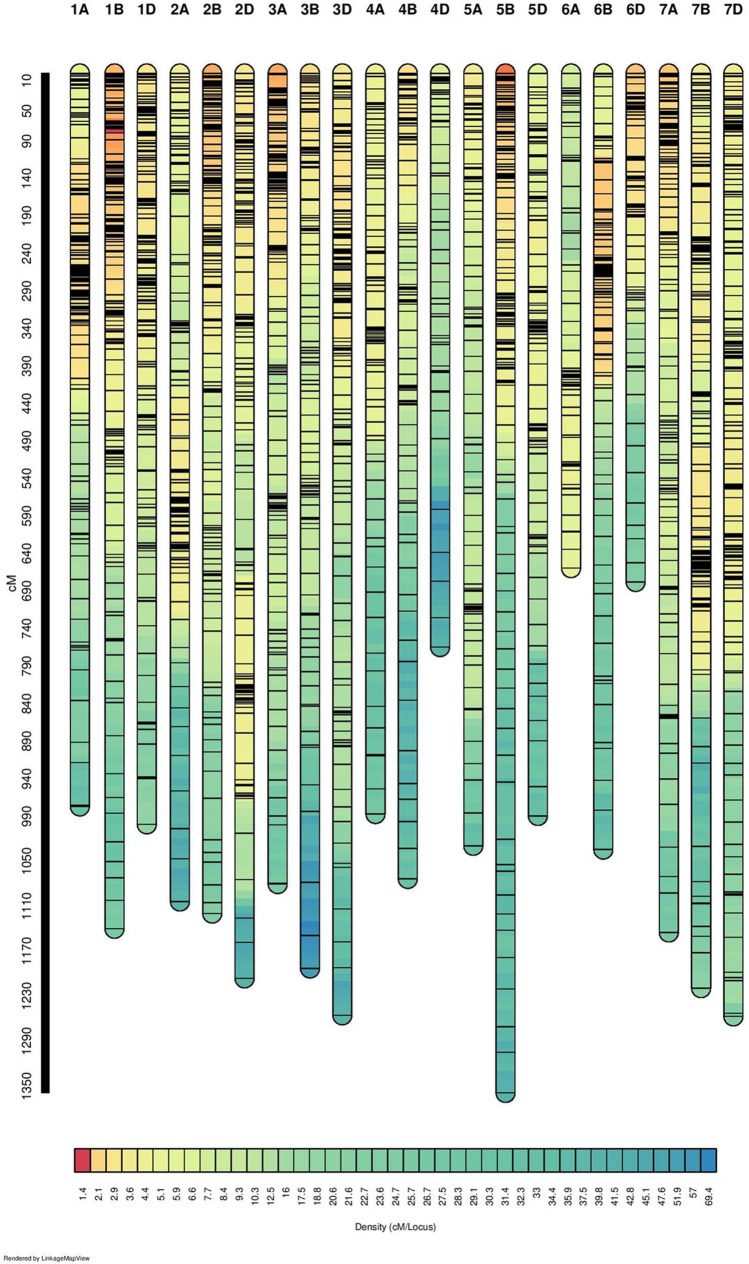
Linkage map constructed from SNP and SSR genotyping in a recombinant inbred population derived from a cross between NI5439 and HD2012.

The genetic linkage map was constructed using a mapping population of 92 RILs derived from the cross between NI5439 and HD2012. A total of 3661 markers comprising of 3589 SNP and 72 SSR markers were utilized for construction of linkage map (Fig. 1, Supplementary Figure S1). The linkage map spanned 22,275.01 cM in length distributed across the 21 wheat chromosomes (Table 1). Probe set ID, marker allele for each genotype, affymetrix SNP ID, reference chromosome, physical position (bp) and sequence details for the filtered 3589 SNPs are given in Supplementary data sheet S1. Average distance between two linked markers ranged from 4.27 cM (1B) to 14.43 cM (4D) with an overall marker interval of 6.08 cM. Marker density ranged from 0.07 cM per marker (4D) to 0.23 cM per marker (1B) with an average of 0.17 cM per marker. Genome wise length analysis of map revealed that A, B, and D genomes spanned 6982.80 cM (31.34%), 8139.80 cM (36.54%), and 7152.42 cM (32.10%), respectively. The number of markers varied from 53 in 4D chromosome to 267 in 1B chromosome. The shortest chromosome was 6A which harbored 96 markers with a genetic length of 659.43 cM. The longest chromosome was 5B which had 220 markers with a genetic length of 1359.04 cM. A genome and D genome had 0.16 cM marker density with 1103 and 1125 total markers respectively, as compared to B genome which has marker density of 0.18 cM per marker with 1433 markers.

**Table 1:** Marker statistics of linkage map constructed from recombinant inbred lines derived from NI5439/HD2012.

Chr	No. of markers	Chromsome length (cM)	%Marker	Average marker interval (resolution)	Marker density
1A	192	977.08	5.24	5.09	0.20
1B	267	1140.19	7.29	4.27	0.23
1D	182	1001.05	4.97	5.50	0.18
2A	164	1103.75	4.48	6.73	0.15
2B	237	1119.90	6.47	4.73	0.21
2D	228	1206.40	6.23	5.29	0.19
3A	205	1080.63	5.60	5.27	0.19
3B	150	1193.09	4.10	7.95	0.13
3D	191	1255.56	5.22	6.57	0.15
4A	128	987.20	3.50	7.71	0.13
4B	126	1073.67	3.44	8.52	0.12
4D	53	764.73	1.45	14.43	0.07
5A	120	1029.56	3.28	8.58	0.12
5B	220	1359.04	6.01	6.18	0.16
5D	128	989.77	3.50	7.73	0.13
6A	96	659.43	2.62	6.87	0.15
6B	209	1034.56	5.71	4.95	0.20
6D	137	677.88	3.74	4.95	0.20
7A	198	1145.15	5.41	5.78	0.17
7B	224	1219.35	6.12	5.44	0.18
7D	206	1257.04	5.63	6.10	0.16
A genome	1103	6982.80	30.13	6.33	0.16
B genome	1433	8139.80	39.14	5.68	0.18
D genome	1125	7152.42	30.73	6.35	0.16
Total	3661	22,275.01		6.08	0.17

### Phenotypic evaluation

The NI5439 × HD2012 derived RIL population showed significant variation for leaf rolling. The descriptive statistical parameters and frequency distribution over all the three environments for leaf rolling is shown in Supplementary Table S1 and Supplementary Figure S2. The mapping population was characterized by average mean value of lines varying from 2.00 to 5.00. Coefficient of variation (CV) ranged from 26.11 to 32.20 with an average CV of 27.82. Standard deviation (SD) ranged from 0.63 to 0.80 with average SD of 0.68. Positive correlation was observed within the replicates of each environment and within each environment for leaf rolling trait (Supplementary Tables S2–S3). Analysis of variance showed significant differences among genotypes of the RIL population in all 3 years (Supplementary Table S4). RILs showed extreme values for leaf rolling than that of parents across all the environments signifying transgressive segregation. This indicates polygenic inheritance of the trait and suggests that alleles with positive effects are distributed among the parents. Mean heritability of 0.94 was observed for leaf rolling trait (Supplementary Table S4).

### QTL identification for leaf rolling

**Figure 2:**
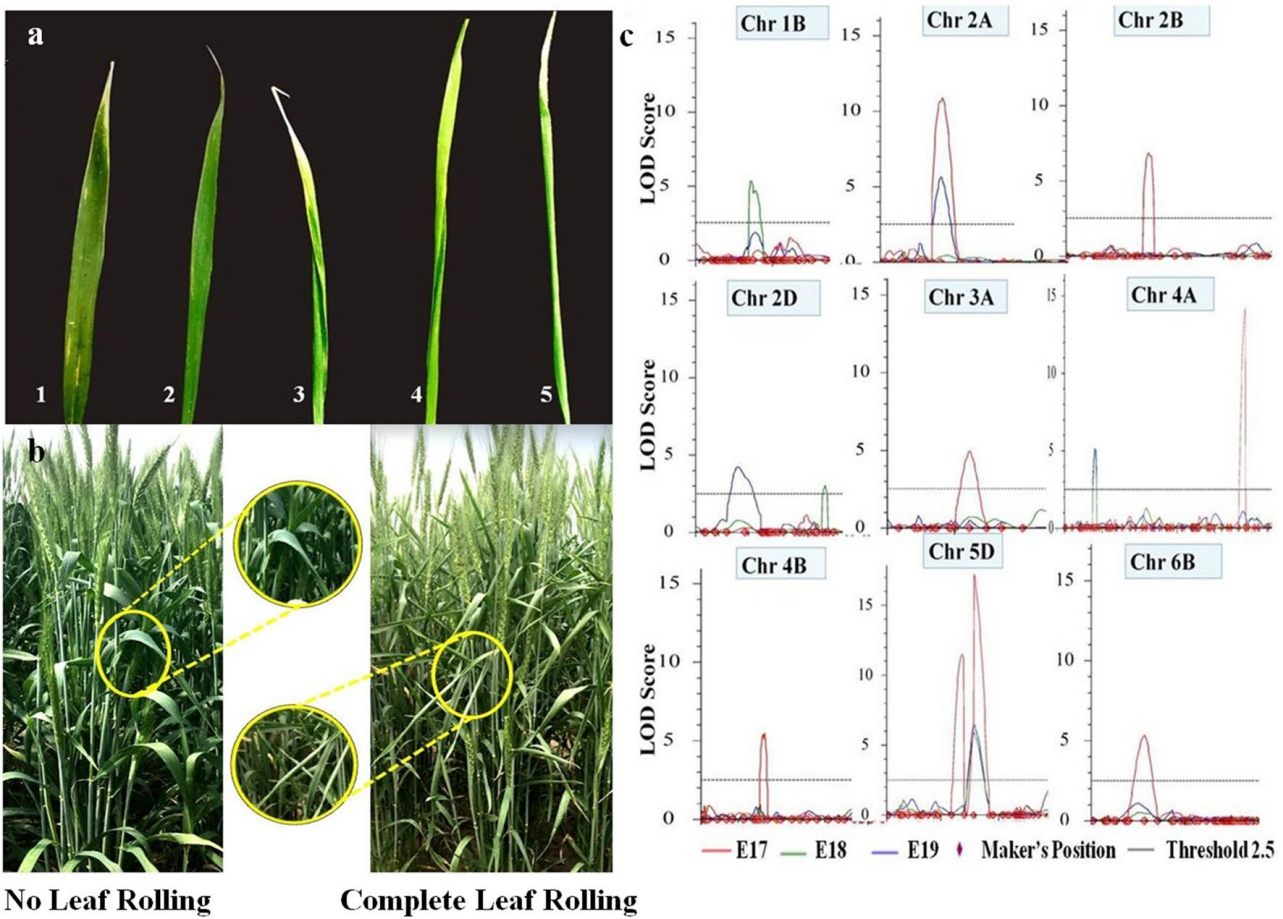
(**a**) Scoring of leaf rolling trait ranging from 1 (no leaf rolling) to 5 (complete leaf rolling); (**b**) RILs of NI5439 × HD2012 mapping population showing difference in leaf rolling from no rolling of the leaf to complete rolling; (**c**) graphical illustration of QTLs detected in three environments for leaf rolling. Red, green and blue represent the three environments E17, E18 and E19.

**Figure 3:**
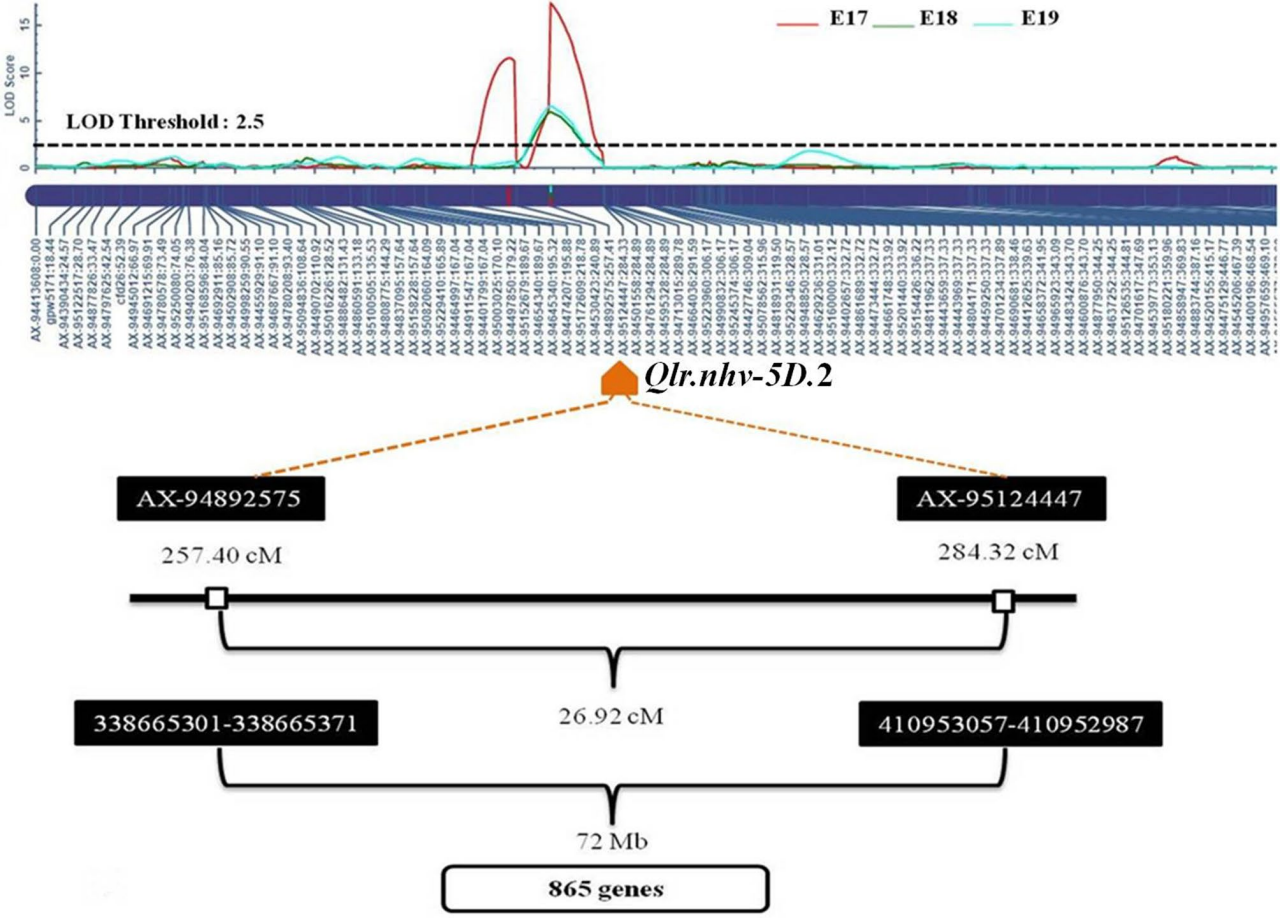
Stable QTL (*Qlr.nhv-5D.2*) for leaf rolling on 5D chromosome during E17 (red), E18 (green) and E19 (blue). Genetic and physical map location of *Qlr.nhv-5D.2* flanked by markers *AX-94892575*- *AX-95124447*.

For the leaf rolling trait, a total of 12 QTLs were detected under drought stress condition during all 3 years (Fig. 2c; Table 2). LOD scores of these QTLs ranged from 3.01 to 17.19. Phenotypic variance explained (%) varied from 4.06 to 20.10%. QTLs identified were associated with chromosomes 1B, 2A, 2B, 2D, 3A, 4A, 4B, 5D and 6B. Eight QTLs were identified during 2017–2018 (E17), whereas, four QTLs were detected during 2018–2019 (E18) and 2019–2020 (E19), with all QTLs showing LOD score greater than 3. On 5D chromosome, a stable QTL *Qlr.nhv-5D.2* was detected consistently throughout all the environments (Fig. 3). *Qlr.nhv-5D.2* was flanked by markers *AX-94892575* and *AX-95124447* (5D:338665301–5D:410952987). In E17, the QTL *Qlr.nhv-5D.2* showed LOD score of 17.19 explaining phenotypic variance of 20.10%. In E18, the QTL *Qlr.nhv-5D.2* showed LOD score 5.84 explained 16.08% of phenotypic variance. While, in E19, the QTL *Qlr.nhv-5D.2* showed LOD score 6.45 and explained 14.25% of phenotypic variance. Another QTL *Qlr.nhv-5D.1* was also detected on 5D chromosome only in E17. On chromosome 2A, QTL *Qlr.nhv-2A* was detected in two environments (E17 and E19). *Qlr.nhv-2A* was flanked by markers *AX-94942225*–*AX-95254393* having LOD score 10.85 and PVE 12.03% in E17, whereas in E19 *Qlr.nhv-2A* explained LOD score 5.64 and PVE 12.13%. On chromosome 4A, QTL *Qlr.nhv-4A.2* was detected in E18 and E19. *Qlr.nhv-4A.2* was flanked by markers *AX-95212081*–*AX-94470023* having LOD score 5.13 and PVE 13.43% in E18 and LOD score of 5.06 and PVE 11.38% in E19. In E17, another QTL *Qlr.nhv-4A.1* flanked by markers *AX-94739181*–*AX-94500554* (LOD score 14.18 and PVE 15.26%) was also identified. On chromosome 2D, two distinct QTLs were detected in E18 and E19. *Qlr.nhv-2D.1* was flanked by markers *AX-94743285*–*AX-94823535* having LOD score 3.01 and PVE 7.44%. On the other hand, *Qlr.nhv-2D.2* was flanked by markers *AX-94661194*–*AX-94603691* having LOD score 4.21 and PVE 15.80%. On rest of the chromosomes viz., 1B (*Qlr.nhv-1B*), 2B (*Qlr.nhv-2B*), 3A (*Qlr.nhv-3A*), 4B (*Qlr.nhv-4B*), 6B (*Qlr.nhv-6B*) QTLs were detected only during a single year.

**Table 2:** QTLs for leaf rolling in RIL mapping population of NI5439 and HD2012.

Chr	QTL	Env	Pos	Flanking markers	LOD	PVE(%)	Add
1B	*Qlr.nhv-1B*	E18	172	*AX-95149749-gwm153*	5.32	15.35	− 0.26
2A	*Qlr.nhv-2A*	E17	681	*AX-94942225–AX-95254393*	10.85	12.03	− 0.32
		E19	679	*AX-94942225–*AX-95254393	5.64	12.13	− 0.27
2B	*Qlr.nhv-2B*	E17	686	*AX-94711931–AX-95260437*	6.86	6.49	− 0.23
2D	*Qlr.nhv-2D.1*	E18	430	*AX-94743285–AX-94823535*	3.01	7.44	− 0.18
	*Qlr.nhv-2D.2*	E19	283	*AX-94661194–AX-94603691*	4.21	15.80	− 0.31
3A	*Qlr.nhv-3A*	E17	823	*AX-94598770–AX-94844071*	4.95	4.06	0.18
4A	*Qlr.nhv-4A.1*	E17	482	*AX-94739181–AX-94500554*	14.18	15.26	0.36
	*Qlr.nhv-4A.2*	E18	80	*AX-95212081–AX-94470023*	5.13	13.43	0.24
		E19	83	*AX-95212081–AX-94470023*	5.06	11.38	0.26
4B	*Qlr.nhv-4B*	E17	262	*AX-94993235–AX-94508980*	5.44	5.43	− 0.21
5D	*Qlr.nhv-5D.1*	E17	237	*AX-95172609–AX-94530423*	11.47	14.01	0.35
	*Qlr.nhv-5D.2*	E17	258	*AX-94892575–AX-95124447*	17.19	20.10	− 0.41
		E18	258	*AX-94892575–AX-95124447*	5.84	16.08	− 0.26
		E19	258	*AX-94892575–AX-95124447*	6.45	14.25	− 0.29
6B	*Qlr.nhv-6B*	E17	142	*AX-94500240–AX-94980566*	5.31	4.47	− 0.19

### Candidate gene identification and in-silico gene expression

A stable QTL for leaf rolling *Qlr.nhv-5D.2* was located on 5D chromosome, flanked by markers *AX-94892575* and *AX-95124447*. Putative gene(s) for leaf rolling was predicted in this marker interval (Fig. 3). Out of 865 genes present in the interval, 14 protein coding genes related to drought tolerance in *T. aestivum* were identified viz., Chlorophyll a/b binding protein, Potassium transporter, HVA22-like protein, Glycosyltransferase, Patatin, Histone proteins: Histone H2A and Histone H4 variant TH011, Peroxidase gene, Dirigent protein, Calcium-transporting ATPase, Auxin efflux carrier component, MADS box protein, 3-ketoacyl-CoA synthase, and heat shock protein 90 (Table 3). Gene ontology (GO) term analysis was carried out for these 14 genes and data is furnished in the Supplementary Table S5. Gene expression of these predicted 14 potential candidate genes under stress and non stress treatments was analyzed in-silico using the wheat expression browser (Supplementary Figure S3). Out of the 14 genes, *TraesCS5D02G238300*,* TraesCS5D02G268000*,* TraesCS5D02G240900* and *TraesCS5D02G249800* were expressed equally throughout all treatments indicating that they are not dependent on the stress/no stress stimulus for their expression. Low to no expression was observed for genes,* TraesCS5D02G293300* and *TraesCS5D02G294500* in all three treatments. The genes, *TraesCS5D02G248600*,* TraesCS5D02G252700*,* TraesCS5D02G308500* and *TraesCS5D02G316200* showed different levels of expression in control treatment but showed low to no expression in the other two treatments. The two genes *TraesCS5D02G240200* and *TraesCS5D02G256400* were up-regulated in drought stress treatment as compared to both the control treatments. *TraesCS5D02G284100* showed similar expression in no stress control and drought stress and displayed slightly lesser expression in the PEG control. While *TraesCS5D02G309500* showed almost similar expression in no stress control and drought stress and slightly higher expression in the PEG control. Based on the in-silico gene expression analysis, six genes exhibited differential expression in no stress control and drought stress treatments. Out of these six genes, four genes viz., *TraesCS5D02G248600*,* TraesCS5D02G252700*,* TraesCS5D02G308500* and *TraesCS5D02G316200* showed down-regulation and two genes viz., *TraesCS5D02G240200* and *TraesCS5D02G256400* showed up-regulation under drought stress. Hence out of the 14 genes these six may be the best candidate genes.

**Table 3:** Candidate gene(s) identified related to drought stress in the marker interval region of QTL *Qlr.nhv-5D.2*.

S. no.	Gene stable ID	Gene description	Source	Gene bp start	Gene bp end
1	*TraesCS5D02G238300*	Chlorophyll a-b binding protein, chloroplastic	UniProtKB/TrEMBL;Acc:W5FYI0	346636682	346638071
2	*TraesCS5D02G240200*	Potassium transporter	UniProtKB/TrEMBL;Acc:A0A1D6S5W8	348479747	348485739
3	*TraesCS5D02G240900*	HVA22-like protein	UniProtKB/TrEMBL;Acc:A0A096UT86	349934068	349935316
4	*TraesCS5D02G248600*	Glycosyltransferase	UniProtKB/TrEMBL;Acc:A0A1D5ZMZ8	355607639	355609171
5	*TraesCS5D02G249800*	Patatin	UniProtKB/TrEMBL;Acc:W5FVZ6	356200779	356204674
6	*TraesCS5D02G252700*	Histone H2A	UniProtKB/TrEMBL;Acc:A0A1D5ZZ26	358798314	358798998
7	*TraesCS5D02G256400*	Peroxidase	UniProtKB/TrEMBL;Acc:A0A1D5ZID1	362661987	362665323
8	*TraesCS5D02G268000*	Heat shock protein 90	UniProtKB/TrEMBL;Acc:Q0Q0I7	371469091	371472885
9	*TraesCS5D02G284100*	Calcium-transporting ATPase	UniProtKB/TrEMBL;Acc:W5FZP2	384852906	384863954
10	*TraesCS5D02G293300*	Auxin efflux carrier component	UniProtKB/TrEMBL;Acc:A0A1D5ZW76	390504143	390506578
11	*TraesCS5D02G294500*	MADS box protein	UniProtKB/TrEMBL;Acc:Q718F3	391729992	391737307
12	*TraesCS5D02G308500*	Histone H4 variant TH011	UniProtKB/Swiss-Prot;Acc:P62785	405334206	405334791
13	*TraesCS5D02G309500*	Dirigent protein	UniProtKB/TrEMBL;Acc:A0A1D5ZQ97	406461906	406462620
14	*TraesCS5D02G316200*	3-Ketoacyl-CoA synthase	UniProtKB/TrEMBL;Acc:A0A1D5ZJN8	410200495	410202372

### Homology modeling of leaf rolling genes in wheat and rice

**Figure 4:**
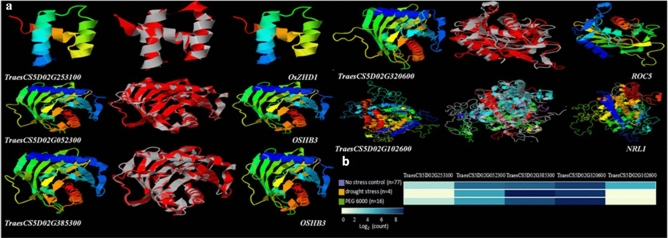
(**a**) 3D protein structures of the predicted leaf rolling genes in wheat (left) along with the protein structure of corresponding gene in rice (right) and their superimposed (middle) 3D structure in red and grey color. Red color represents wheat protein whereas grey represents rice protein. (**b**) Expression profiles of predicted leaf rolling wheat genes in three different conditions (no stress control, drought stress and PEG 6000). The dark and light intensity of the blue color represents the higher and lower relative abundance of the transcript.

In maize and rice, extensive studies have been undertaken for leaf rolling as a component trait of drought tolerance. We compared the protein sequences of leaf rolling genes with the 5D chromosome of wheat reference genome to identify putative genes in wheat. In case of maize, three reported genes viz., *Lbl1* (*leafbladeless1*), *Rld1* (*Rolled leaf1*), *ZmOCL5* (*Outer Cell Layer5*) were used for comparison. However, none of them showed more than 80% identity in the prescribed QTL interval. In case of rice, 17 genes related to leaf rolling viz., *YABBY1*,* COW1 (CONSTITUTIVELY WILTED1)/NAL7 (NARROW LEAF7)*,* OsHB1*,* ADL1 (ADAXIALIZED LEAF1)*,* SSL1 (SHALLOT-LIKE1)/RL9*,* NRL1 (NARROW AND ROLLED LEAF1)*,* ACL1 (Abaxially Curled Leaf1)*,* ACL2*,* LC2*,* Roc5 (Rice outermost cell-specific gene5)*,* SRL1 (SEMI-ROLLED LEAF1)*,* RL14*,* OsZHD1*,* REL1 (Rolled and Erect Leaf1)*,* REL2*,* LRRK1*,* OsHB3* were utilized for comparison. We identified five genes in wheat showing more than 80% identity with four reported genes in rice viz., *NRL1*,* OsZHD1*,* Roc5*, and *OsHB3* (Table 4). Out of these, one gene (*TraesCS5D02G253100*) falls exactly in the QTL interval (338665301–410952987) and is showing 96.9% identity with *OsZHD1*. Two genes similar to *OsHB3* were identified viz. *TraesCS5D02G052300* and *TraesCS5D02G385300* exhibiting 85.6% and 91.8% identity, respectively. One gene *TraesCS5D02G320600* was having 83.9% identity with *Roc5* gene. Lastly, *TraesCS5D02G102600* showed 100% identity with *NRL1* gene. The last four genes did not fall in the exact QTL interval. Nevertheless, they are reported on 5D chromosome near to the interval. The 3D protein structure of these five wheat genes was predicted and superimposed on the respective protein from rice (Fig. 4A). The 3D protein structure of all the five proteins was found to be significantly similar to the corresponding rice protein. The physiochemical properties of these proteins were also predicted, and data is furnished in Supplementary Table S6.

**Table 4:** Homology modeling of Leaf rolling genes in Wheat and Rice.

S. no	Rice genes	Gene hit	Genomic location	Query %	E-val	Identity (%)	Subcellular location	P value	Raw score
1	*OsZHD1*	*TraesCS5D02G253100*	5D: 359474816–359475706	60.2	3.60E−38	96.9	Nucleus	0.00E+00	168.00
2	*OsHB3*	*TraesCS5D02G052300*	5D: 50483246–50488859	75.4	0	85.6	Nucleus	0.00E+00	620.69
3		*TraesCS5D02G385300*	5D: 454414059–454419771	48.4	0	91.8	Chloroplast	0.00E+00	550.73
4	*ROC5*	*TraesCS5D02G320600*	5D:412737994–412744775	75.8	8.20E−79	83.9	Nucleus	1.52E−12	359.98
5	*NRL1*	*TraesCS5D02G102600*	5D:115947470–115952140	80.8	3.00E−30	100.0	Chloroplast outer membrane	1.64 E -08	1134.16

## Discussion

Leaf rolling is an important drought tolerance mechanism having polygenic control with additive effects which has been reported in various crops like wheat, rice, maize, and sorghum^27^. In a study on genetics of leaf rolling, involvement of polygenes with additive effects was reported in wheat^27^. Another study in tetraploid wheat *T. turgidum* ssp. *dicoccoides* (AABB, 2n = 4x = 28) reported eleven QTLs associated with leaf rolling, located on chromosomes 1A, 2A, 2B, 4B, 5A, 5B, 6A, 6B, 7A, and 7B^24^. Five of these QTLs significantly co-localized with QTLs associated with plant productivity. In rye (RR, 2n = 14), a distant relative of wheat, four stable QTLs for flag leaf rolling located on chromosomes 3R, 5R, and 7R were reported^25^. However, only few studies on leaf rolling is reported in hexaploid wheat. Leaf rolling was found out to be a trait showing non-Mendelian inheritance. Frequency distribution of leaf rolling in the mapping population showed a skewed normal distribution with large number of RILs showing lesser values of leaf rolling score under drought stress. The trait displayed high heritability and significant positive correlation within the replicates of each environment and within each environment which shows its stability. Mapping population showed significant genotypic variation. Transgressive segregation was evident since RILs showed extreme values for leaf rolling than that of parents across all the environments. All the above points suggest polygenic inheritance of leaf rolling trait. In order to dissect complex polygenic traits like yield a reductionist approach is successful where the complex trait is partitioned into several component traits^28^. Drought tolerance is also a complex trait whose inheritance is hard to follow. Hence, we have studied a component trait viz., leaf rolling for quantifying drought tolerance. Bi-parental RIL populations are best suited for studying polygenic traits which when combined with genome-wide molecular markers make pin pointing QTLs more efficient in a polyploid crop like wheat. In the current study, 35 K Axiom Wheat Breeder’s SNP Array and microsatellite markers were utilized for mapping of QTLs for flag leaf rolling. A genetic map was constructed using 3661 markers distributed across 21 wheat chromosomes. The 35 K wheat array provided sufficient number of polymorphic markers to construct genetic map and to identify potential putative QTLs. QTL mapping in the RIL population identified 12 QTLs for leaf rolling trait under drought condition in three consecutive years. These QTLs were associated with chromosomes 1B, 2A, 2B, 2D, 3A, 4A, 4B, 5D and 6B. Interestingly, a stable QTL *Qlr.nhv-5D.2* was identified on 5D chromosome with a common marker interval (*AX-94892575–AX-95124447*) indicating that the putative genes related to leaf rolling/drought tolerance are residing in this interval.

In the physical interval 5D:338665301–5D:410952987, 14 putative genes related to drought tolerance were identified. *In-silico* gene expression analysis showed varied expression of these genes under three treatments viz., no stress control, drought stress and PEG6000. Four genes viz., *TraesCS5D02G238300*,* TraesCS5D02G268000*,* TraesCS5D02G240900* and *TraesCS5D02G249800* displayed equal expression across treatments which ruled out their possibility to be candidate genes. The lack of expression of two genes viz., *TraesCS5D02G293300* and *TraesCS5D02G294500* in all three treatments also ruled out their possibility to be candidate genes. Other two genes *TraesCS5D02G284100* and *TraesCS5D02G309500* can also be ruled out since they showed similar expression in no stress control and drought stress while slight variation under PEG control. Hence, out of 14 genes only six are qualified to be the potential candidate genes since they showed differential expression under drought and no stress conditions. Out of these six genes, two genes *TraesCS5D02G240200* and *TraesCS5D02G256400* are up-regulated under drought stress. The first gene, a Potassium transporter gene (*TraesCS5D02G240200*) is reported to be a positive regulator (*OsHAK1*-High Affinity Potassium Transporter) of drought tolerance in rice^29^. *OsHAK1* overexpression plants were shown to be yielding 35% more than wild type plants under water stress conditions. The second gene *TraesCS5D02G256400* is the Peroxidase gene. Under abiotic stress the principal defensive mechanism at cell level exhibited by plants is an increase in the reactive oxygen species (ROS) viz., hydroxyl radical, singlet oxygen, superoxide and hydrogen peroxide (H_2_O_2_)^30^. Excessive production of ROS has its side effects leading to protein degradation, enzyme inhibition, DNA and RNA damage, cellular damage and finally cell death. This makes ROS detoxification important for protecting plant cell from the toxic impact of ROS. It is achieved by enzymatic and non-enzymatic antioxidant elements. One of the enzymatic antioxidants is peroxidase. In a study involving wild relatives of wheat, the expression of peroxidases was found to be higher under drought stress^31^. These evidences prove that both the genes are actively involved in drought tolerance and positively regulates the plant’s response to drought stress.

The four genes viz., *TraesCS5D02G248600*,* TraesCS5D02G252700*,* TraesCS5D02G308500* and *TraesCS5D02G316200* were shown to be down-regulated under drought stress and PEG treatments. The gene *TraesCS5D02G248600* is shown to have Glycosyltransferase activity. Uridine diphosphate (UDP)-glycosyltransferases (UGTs) are group of enzymes which are involved in transferring sugar moieties onto an array of small molecules and regulate various metabolic pathways^32^. Studies in Arabidopsis showed that glycosyltransferase genes viz., *UGT79B2* and *UGT79B3*^32^ and *AtUGT76C2*^33^ were induced by abiotic stresses like cold, salt and drought stresses. The two genes *TraesCS5D02G252700* and *TraesCS5D02G308500* identified in our study were histone related genes, Histone H2A and Histone H4 variant TH011, respectively. This clearly points to the chromatin changes occurring under drought stress conditions^34^. An abiotic stress responsive H2A variant gene *TaH2A*.7 was reported in wheat which enhanced drought tolerance and promotes stomatal closure when overexpressed in Arabidopsis while it had no effect on the response to saline, osmotic and oxidative stresses^35^. Histone acetylation is reported to be correlated with drought stress and ABA responses in plants^34^. In rice, induction of *HAT* (Histone acetyltransferase) genes enhanced acetylation of histone molecules including H4 under drought stress conditions^36^. *TraesCS5D02G316200* is having 3-ketoacyl-CoA synthase activity. 3-ketoacyl-CoA synthase (KCS) genes are involved in the biosynthesis of cuticular wax which forms the first protective layer of plants^37^. Hence, cuticular wax plays a major role in abiotic and biotic stress tolerance. It is reported that the total amount of wax per unit leaf area increased by 80% in plants under water stress than control. In wheat, glaucous genotypes have a significantly higher yield than non-glaucous in normal and moderate drought environments^38^. A similar positive correlation has been observed in barley^39^. *OsWSL1* is one of the 3-ketoacyl-CoA synthase (KCS) genes in rice which is related to drought tolerance since *Oswsl1* mutant exhibited drought sensitivity^40^. *AtCER6* is another KCS gene characterized in Arabidopsis which when transformed in tomato (transgenic) lines showed significant increase in water use efficiency (WUE) and enhanced drought tolerance as compared to wild type control^41^. Even though these genes are associated with drought tolerance, none of them are directly linked to leaf rolling. Hence we have compared reported leaf rolling genes in rice and maize with the wheat genome sequence.

Rolled leaf trait is well studied in rice where it is one of the breeding objectives for genetic improvement. This trait provides additional benefits like reduced water loss by transpiration, thus imparting drought tolerance. In rice, around 70 genes/QTLs for rolled leaf trait have been reported^22, 23^. Some of the cloned genes are *YABBY1*^42^,* COW1/NAL7*^43, 44^,* OsHB1*,* OsHB3 *and* OsHB5*^45^,* ADL1*^46^,* SSL1/RL9*^47^,* NRL1*^48^,* ACL1* and *ACL2*^49^,* LC2*^50^,* Roc5*^51^,* SRL1*^52^,* RL14*^53^,* OsZHD1* and *OsZHD2*^54^, *REL1*^55^,* REL2*^56^,* OsARVL4*^57^,* LRRK1*^58^*.* We have utilized 17 of these genes for comparative studies. Comparative homology modeling and the 3D protein structure superimposition of leaf rolling rice genes with wheat revealed five important genes in 5D chromosome of wheat genome. These five genes exhibited more than 80% identity with four reported genes in rice viz., *NRL1*,* OsZHD1*,* Roc5*, and *OsHB3.* Out of the five genes, only *TraesCS5D02G253100* (5D: 359474816–359475706) falls exactly within the QTL interval region of *Qlr.nhv-5D.2* (5D:338665301-5D:410952987)*. TraesCS5D02G253100* has 96.9% structure similarity to *OsZHD1*, which is zinc finger homeodomain class homeobox transcription factor (TF). Studies in most of the cloned leaf rolling genes in rice, the involvement of bulliform cells in regulation of rolling has been confirmed. For instance, the expression of the genes viz., *Roc5* and *OsZHD1* regulate the number, size and/or arrangement of bulliform cells leading to adaxial/abaxial rolling of leaf^15^ while *OsHB3* controlled leaf rolling by affecting leaf polarity^46^. Having close structural similarity to *OsZHD1*, *TraesCS5D02G253100* might be responsible for increasing the no. of bulliform cells leading to abadixal leaf rolling in wheat^54^. Similarly, other identified genes viz. *TraesCS5D02G052300*,* TraesCS5D02G385300*,* TraesCS5D02G320600* and *TraesCS5D02G102600* might have the same function as of their respective genes in rice *OsHB3*, *ROC5* and *NRL1*. In-silico expression profiling patterns of the five genes were predicted under drought stress as compared to no stress control and PEG treatment (Fig. 4b). Expression of *TraesCS5D02G253100* was reduced under drought stress as compared to both the controls. While the expression of *TraesCS5D02G052300* decreased under drought stress and PEG treatment. Under drought stress expression of *TraesCS5D02G385300* was higher than the no stress control. The gene *TraesCS5D02G102600* was not at all expressed under drought stress and PEG treatment. Hence, there is differential expression of these four genes under drought and no stress conditions. Whereas, in case of *TraesCS5D02G320600* expression was similar in all the treatments. Since *TraesCS5D02G253100* (Zn finger homeodomain class homeobox TF) shows > 95% structure similarity to *OsZHD1*, falls exactly in the QTL interval *Qlr.nhv-5D.2* and shows differential expression under drought stress and no stress condition, it could be the best candidate gene for leaf rolling which needs further functional validation across diverse genotypes.

## Conclusion

In the present study, a linkage map of RIL population from the cross NI5439 × HD2012 spanning 22,275.01 cM using 3589 SNPs and 72 SSR was constructed. This linkage map was utilized to identify QTLs for leaf rolling under moisture stress condition. Twelve QTLs were detected on chromosomes viz., 1B, 2A, 2B, 2D, 3A, 4A, 4B, 5D and 6B. A stable QTL *Qlr.nhv-5D.2* was identified on 5D chromosome. Marker assisted breeding can be employed for transfer of this QTL to improve drought tolerance in wheat cultivars. Six best putative candidate genes associated with drought tolerance were identified within the QTL interval on 5D. Moreover, five genes in wheat showing more than 80% identity with four reported leaf rolling genes in rice viz., *NRL1*,* OsZHD1*,* Roc5*, and *OsHB3* were also identified. Out of these, *TraesCS5D02G253100* coding for Zn finger homeodomain class homeobox transcription factor located within the QTL interval *Qlr.nhv-5D.2* could be the best candidate gene for leaf rolling. Further investigation can validate the exact gene for map-based cloning, understanding the molecular basis of leaf rolling and utilization for improvement of drought tolerance in wheat.

## Material and methods

### Plant material

Mapping population consisted of 92 F_2:16_ RILs developed from cross between genotypes NI5439 and HD2012 by single spike descent method^59^. NI5439 is a variety released for cultivation in the peninsular region of India with parentage REMP 80/3* NP 710. HD2012 is a variety developed from the cross HD1467/HB208.

### Experimental design and phenotyping

The experiment was conducted during 2017–2018, 2018–2019 and 2019–2020 growing seasons at Indian Agriculture Research Institute, New Delhi (28.6377° N, 77.1571° E). Ninety-two RILs along with parental lines were analyzed in a randomized complete block design (RCBD) in two replications. The experimental unit consisted of a four-row plot of 1 m length and 25 cm row to row distance. The RILs were given irrigation after 21 days of sowing in order to avoid any crop failure, after which no irrigation was provided. Phenotyping of flag leaf rolling was visually scored after the flowering stage on the scale from 1 (no leaf rolling) to 5 (complete leaf rolling) (Fig. 2a,b). The three environments i.e. 2017–2018, 2018–2019 and 2019–2020 hereafter will be mentioned as E17, E18 and E19, respectively.

### Statistical analysis

Descriptive statistical parameters analysis and correlation studies were conducted using Statistica 13.0 software package (Stat-Soft, Inc., USA, https://www.statsoft.com). ANOVA and Heritability of the trait was calculated using the AOV functionality of QTLIcimapping version 4.2. The following model was used in ANOVA (1) and estimation of heritability per mean (2).

1 $$y_{ik} = \, \mu \, + b_{k \, + } g_{i} + \varepsilon_{{{\text{ik}}}} ,\;i = {1}, \ldots ,g;\;k = {1}, \ldots ,r$$

where the number of genotypes is equal to *g*, and the number of blocks is equal to *r*. Assuming *y*_*jk*_ is the oberservation of the *i*th genotype in the *k*th block

2 $${\text{h}}^{{2}} = {\text{ V}}_{{\text{G}}} / \, ({\text{V}}_{{{\text{G }} + }} {1}/{\text{r V}}_{{\text{s}}} ).$$

For QTL mapping Best linear unbiased estimates (BLUE) were calculated using QTL IciMapping software v4.2^60^.

### Genotyping and linkage map construction

High quality genomic DNA of parental genotypes and RILs was isolated using CTAB method^61^. Qualitative and quantitative check was done using 0.8% agarose gel and Nanodrop spectrophotometer. For SSR markers, PCR was performed in reaction volume of 10 μl comprising 1 μl of each primer (5 pmol/μl), 2 μl of genomic DNA (25 ng) and 2 μl of nuclease free water added to 4 μl of reaction mix (Go Green Taq Promega) in 96-well PCR plates with thermal seal in Eppendorf thermal cycler with a thermal profile of initial denaturation step of 94.0 °C for 4 min, followed by 45 cycles of 94.0 °C for 1 min (denaturation), 50–60 °C (annealing temperature depending on primer) for 1 min, 72.0 °C for 1 min (primer extension) and a final extension of 72.0 °C for 10 min and storage at 4.0 °C. The SSRs found to be polymorphic between parents were used for genotyping of 92 RILs. The amplified PCR products of SSR markers were subsequently resolved on 3.5% MetaPhorTM (Lonza) gel in1X TBE buffer. The gel stained in ethidium bromide was visualized under UV-trans-illuminator in a gel documentation system (Syngene G: Box Gel Documentation System).

SNP Genotyping was conducted using Axiom 35 K Breeder’s SNP Array^62^ by Imperial Life Sciences (Gurgaon, India). Genotyping calls for 35,143 SNP markers were obtained, which were filtered in a series of steps. Only those SNPs which showed polymorphism between two parents were utilized for linkage map construction. Out of 7601 polymorphic SNP markers, SNPs lacking any chromosome ID and position were removed. Polymorphic markers having more than 10 missing values for the RILs were also filtered out. For construction of genetic map, only the polymorphic markers with minor allele frequency of more than 0.3 were considered. These markers were tested for significant segregation distortion using Chi-square test. In addition to the selected 4287 SNP markers, 139 polymorphic SSR markers were also used in development of linkage map.

Before linkage map construction, BIN tool algorithm implemented in QTL IciMapping software v4.2^60^ was used for binning of markers having identical segregation patterns. A total of 3661 (3589 SNP + 72 SSR) markers were finally utilized for linkage map construction. Twenty one linkage groups (LGs) were determined based on LOD (logarithm of odds) threshold value greater than 3. The ordering of 3661 markers distributed over 21 chromosomes was performed using SER (SERiation) algorithm implemented in QTL IciMapping software v4.2^63^. Kosambi mapping function was used for conversion of recombination frequencies between markers to centiMorgan. The R package *LinkageMapView* was utilized to graphically display linkage map^64^.

### QTL mapping

QTL mapping was performed using BIP functionality in QTL IciMapping v 4.2 (https://www.isbreeding.net/software/)^60^ employing the Inclusive Composite Interval Mapping of Additive function (ICIM-ADD). The following parameters were used: scanning step was set at 1.00 cM, p value was set at 0.001, minimum 2.50 LOD threshold was set to declare significant QTLs. QTLs detected for leaf rolling were designated according to standard nomenclature^65^.

### Candidate gene prediction

QTL intervals obtained in linkage map were further studied for prediction of candidate gene(s) associated with the respective QTLs using wheat sequence (International Wheat Genome Sequencing Consortium, 2018) available at Ensembl Plants (https://plants.ensembl.org/Triticum_aestivum/Info/Index). Marker intervals were mapped on for their physical locations and sequence between the interval was retrieved using BLAST, homology was selected on basis of E value = 1E−100 and 100% identity. The number and kind of genes present in the sequence were obtained using BioMart tool.

### Homology modeling

Amino acid sequence of already reported genes for leaf rolling in rice and maize were retrieved using RAP-DB (Rice Annotation Project Database; https://rapdb.dna.affrc.go.jp/) and MaizeGDB (Maize Genetics and Genomics database; https://www.maizegdb.org/). Putative wheat genes responsible for leaf rolling present within the QTL interval region were identified by BLASTP search using these sequences against the fully annotated latest release of reference genome (International Wheat Genome Sequencing Consortium, 2018) using Ensembl Plants (https://plants.ensembl.org/Triticum_aestivum/Info/Index). Sequences having E value below zero and identity greater than 80% were selected. The identified wheat genes were then analyzed on ProtParam tool of Expasy website (https://web.expasy.org/protparam/) for the various physiochemical properties. BUSCA (https://busca.biocomp.unibo.it/) was used for predicting the subcellular localization of predicted leaf rolling wheat genes. Protein 3D structure of the wheat genes was predicted using Swiss-Model Server (https://swissmodel.expasy.org/). In order to check significant similarity between the predicted genes and their corresponding genes in rice, the predicted genes were superimposed based on statistical and probability scores and root-square deviation (RMSD) using FATCAT tool (flexible structure alignment by changing fragment pairs allowing twists)^66^. To perform the expression profiling patterns of the identified leaf rolling genes under drought stress RNA-seq expression data was accessed through Wheat expression Browser (https://www.wheat-expression.com/) powered by expression Visualization and Integration Platform (expVIP)^67, 68^.

## Supplementary information

Supplementary Figure 1.

Supplementary Figures.

Supplementary Information.

Supplementary Tables.
