# Supplementary material for: QTL detection and putative candidate gene prediction for leaf rolling under moisture stress condition in wheat
Source: Sci Rep. 2020 Oct 29;10:18696. doi: 10.1038/s41598-020-75703-4 (PMC7596552; doi:10.1038/s41598-020-75703-4)
Supplement: Supplementary file 2 — Supplementary Figures. [file 41598_2020_75703_MOESM2_ESM.docx]

**Supplementary Figures**

**QTL DETECTION AND PUTATIVE CANDIDATE GENE PREDICTION FOR LEAF ROLLING UNDER MOISTURE STRESS CONDITION IN WHEAT**

**Aakriti Verma, M. Niranjana, S.K. Jha, Niharika Mallick, Priyanka Agarwal and Vinod**

*Division of Genetics, ICAR-Indian Agricultural Research Institute, New Delhi-110012, India*

Figure S1. A detailed view of constructed linkage map from SNP and SSR genotyping in a recombinant inbred population derived from a cross between NI5439 and HD2012.


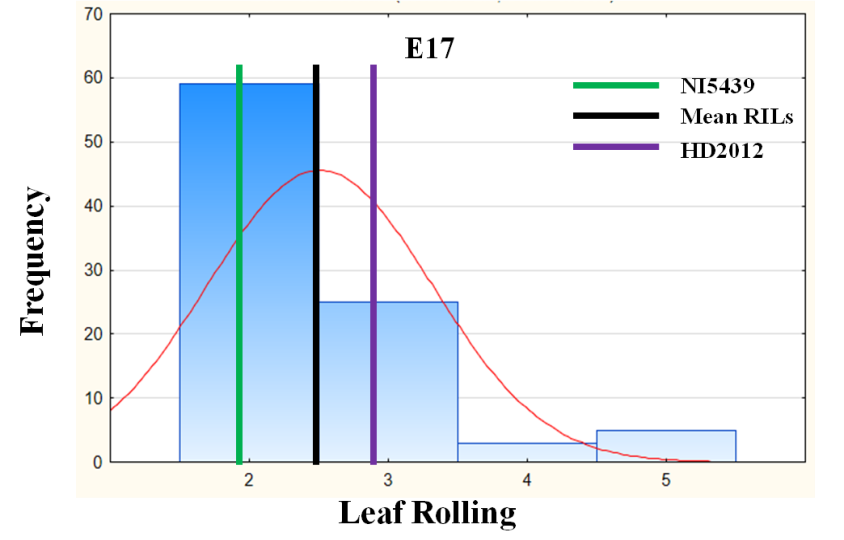

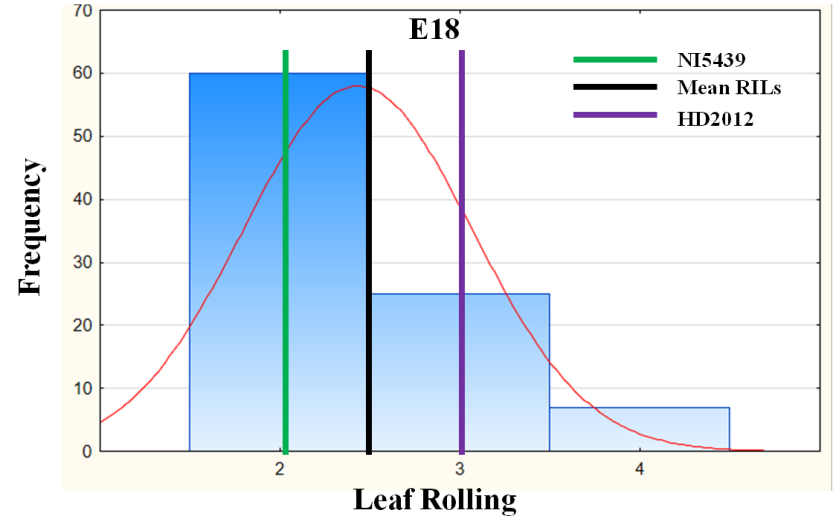

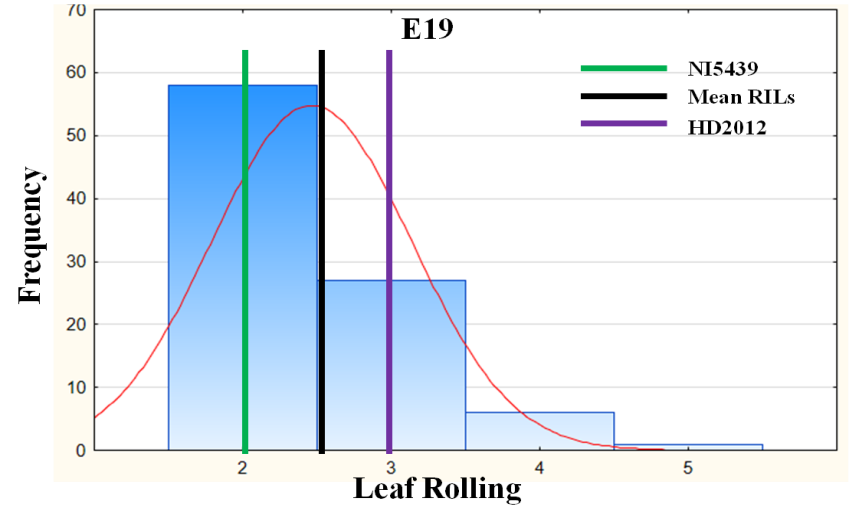


**A**

**B**

**C**

Figure S2. Frequency distribution for Leaf Rolling (LR) evaluated over three environments (A-C). Vertical lines indicate the mean of the parental genotypes NI5439 (green line) and HD2012 (purple line), and RILs (black line)


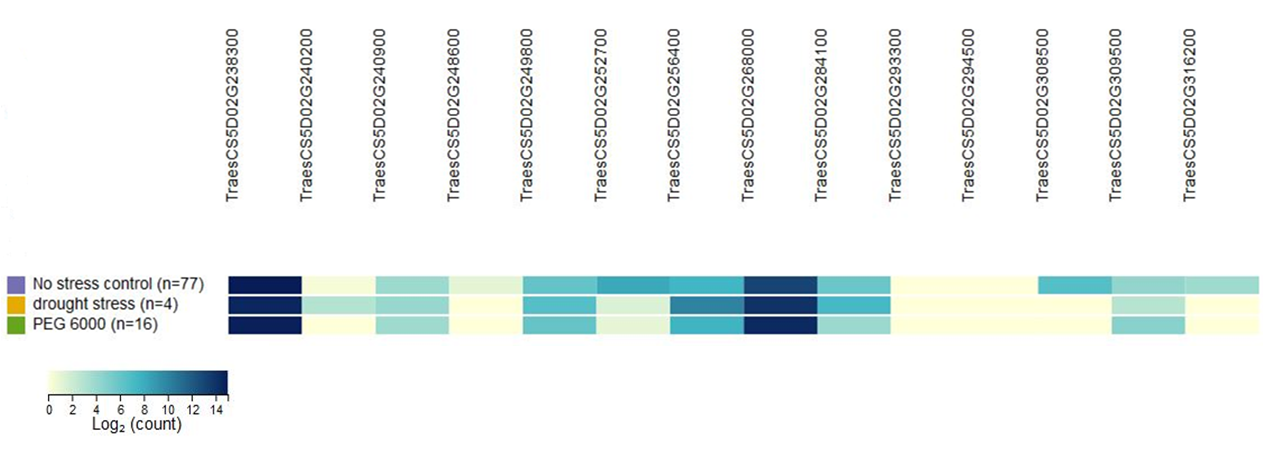


Figure S3. Expression profiles of predicted candidate wheat genes in three different conditions (No stress control, Drought stress and PEG 6000). The dark and light intensity of the blue color represents the higher and lower relative abundance of the transcript

.
