# Supplementary material for: QTL detection and putative candidate gene prediction for leaf rolling under moisture stress condition in wheat
Source: Sci Rep. 2020 Oct 29;10:18696. doi: 10.1038/s41598-020-75703-4 (PMC7596552; doi:10.1038/s41598-020-75703-4)
Supplement: Supplementary file 4 — Supplementary Tables. [file 41598_2020_75703_MOESM4_ESM.docx]

**Supplementary Tables**

**QTL DETECTION AND PUTATIVE CANDIDATE GENE PREDICTION FOR LEAF ROLLING UNDER MOISTURE STRESS CONDITION IN WHEAT**

**Aakriti Verma, M. Niranjana, S.K. Jha, Niharika Mallick, Priyanka Agarwal and Vinod**

*Division of Genetics, ICAR-Indian Agricultural Research Institute, New Delhi-110012, India*

Supplementary Table S1. Phenotypic Summary of Leaf Rolling evaluated at three environments

| **Environments** | **Parents** | | **RILs** | | | | | |
| --- | --- | --- | --- | --- | --- | --- | --- | --- |
|  | **NI5439** | **HD2012** | **Mean ± SE^1^** | **Min** | **Max** | **Variance** | **SD^2^** | **CV^3^** |
| **E17** | 2 | 3 | 2.50 ± 0.08 | 2.00 | 5.00 | 0.64 | 0.80 | 32.20 |
| **E18** | 2 | 3 | 2.42 ± 0.06 | 2.00 | 4.00 | 0.40 | 0.63 | 26.11 |
| **E19** | 2 | 3 | 2.44 ± 0.06 | 2.00 | 5.00 | 0.44 | 0.66 | 27.35 |
| **Average** | 2 | 3 | 2.46 ± 0.07 | 2.00 | 5.00 | 0.47 | 0.68 | 27.82 |

**^1^**Standard error

^2^Standard deviation^3^Coefficient of variation

**Supplementary Table S2. Correlation coefficients of leaf rolling trait among three environments**

| Environment | E17 | E18 | E19 |
| --- | --- | --- | --- |
| E17 | 1 |  |  |
| E18 | 0.808* | 1 |  |
| E19 | 0.897* | 0.886* | 1 |

***Correlations are significant at p < 0.05 N=92**

| Replication | E17-R1 | E18-R1 | E19-R1 |
| --- | --- | --- | --- |
| E17-R2 | 1.000* |  |  |
| E18-R2 |  | 1.000* |  |
| E19-R2 |  |  | 1.000* |

**Supplementary Table S3. Correlation coefficients between replicates in each environment for leaf rolling trait**

***Correlations are significant at p < .05000 N=92**

Supplementary Table S4. Analysis of Variance and Heritability values for Leaf Rolling across three environment

| **Trait Leaf Rolling** | | |
| --- | --- | --- |
| **Source DF MS** | | |
| **Genotype** | 93 | 2.67** |
| **Environment** | 2 | 0.28** |
| **Genotype*Environment** | 186 | 0.15 |
| **Heritability (h^2^)** | 0.94 | |

**Significant at 1% level; DF=Degrees of freedom; MS= Mean square

Supplementary Table S5. Functional Annotation of the candidate genes identified for drought stress in the marker interval of *Qlr.nhv-5D.b*

| **S.No.** | **Gene stable ID** | **GO term accession** | **GO term name** | **GO domain** |
| --- | --- | --- | --- | --- |
| 1. | *TraesCS5D02G238300* | GO:0009535 | chloroplast thylakoid membrane | cellular_component |
|  |  | GO:0009765 | photosynthesis, light harvesting | biological_process |
|  |  | GO:0016168 | chlorophyll binding | molecular_function |
| 2. | *TraesCS5D02G240200* | [GO:0016020](http://amigo.geneontology.org/amigo/term/GO:0016020) | Membrane | cellular_component |
|  |  | [GO:0015079](http://amigo.geneontology.org/amigo/term/GO:0015079) | potassium ion transmembrane transporter activity | molecular_function |
|  |  | [GO:0071805](http://amigo.geneontology.org/amigo/term/GO:0071805) | potassium ion transmembrane transport | biological_process |
| 3. | [*TraesCS5D02G240900*](file:///\\plants.ensembl.org\triticum_aestivum\Gene\Summary?db=core;g=TraesCS5D02G240900) | [GO:0016021](http://amigo.geneontology.org/amigo/term/GO:0016021) | integral component of membrane | cellular_component |
|  |  | [GO:0009737](http://amigo.geneontology.org/amigo/term/GO:0009737) | response to abscisic acid | biological_process |
| 4. | [*TraesCS5D02G248600*](file:///\\plants.ensembl.org\triticum_aestivum\Gene\Summary?db=core;g=TraesCS5D02G248600) | [GO:0016758](http://amigo.geneontology.org/amigo/term/GO:0016758) | transferase activity, transferring hexosyl groups | molecular_function |
| 5. | [*TraesCS5D02G249800*](file:///\\plants.ensembl.org\triticum_aestivum\Gene\Summary%3fdb=core;g=TraesCS5D02G249800) | [GO:0016787](http://amigo.geneontology.org/amigo/term/GO:0016787) | hydrolase activity | molecular_function |
|  |  | [GO:0016042](http://amigo.geneontology.org/amigo/term/GO:0016042) | lipid catabolic process | biological_process |
| 6. | [*TraesCS5D02G252700*](file:///\\plants.ensembl.org\triticum_aestivum\Gene\Summary%3fdb=core;g=TraesCS5D02G252700) | [GO:0005634](http://amigo.geneontology.org/amigo/term/GO:0005634) | Nucleus | cellular_component |
|  |  | [GO:0003677](http://amigo.geneontology.org/amigo/term/GO:0003677) | DNA binding | molecular_function |
| 7. | *TraesCS5D02G256400* | [GO:0004601](http://amigo.geneontology.org/amigo/term/GO:0004601) | peroxidase activity | molecular_function |
|  |  | [GO:0005576](http://amigo.geneontology.org/amigo/term/GO:0005576) | extracellular region | cellular_component |
|  |  | [GO:0055114](http://amigo.geneontology.org/amigo/term/GO:0055114) | oxidation-reduction process | biological_process |
| 8. | [*TraesCS5D02G268000*](file:///\\plants.ensembl.org\triticum_aestivum\Gene\Summary?db=core;g=TraesCS5D02G268000) | [GO:0005524](http://amigo.geneontology.org/amigo/term/GO:0005524) | ATP binding | molecular_function |
|  |  | [GO:0006457](http://amigo.geneontology.org/amigo/term/GO:0006457) | protein folding | biological_process |
| 9. | *TraesCS5D02G284100* | [GO:0016020](http://amigo.geneontology.org/amigo/term/GO:0016020) | Membrane | cellular_component |
|  |  | [GO:0005388](http://amigo.geneontology.org/amigo/term/GO:0005388) | calcium transmembrane transporter activity, phosphorylative mechanism | molecular_function |
|  |  | [GO:0070588](http://amigo.geneontology.org/amigo/term/GO:0070588) | calcium ion transmembrane transport | biological_process |
| 10. | [*TraesCS5D02G293300*](file:///\\plants.ensembl.org\triticum_aestivum\Gene\Summary%3fdb=core;g=TraesCS5D02G293300) | [GO:0016021](http://amigo.geneontology.org/amigo/term/GO:0016021) | integral component of membrane | cellular_component |
|  |  | [GO:0009734](http://amigo.geneontology.org/amigo/term/GO:0009734) | auxin-activated signaling pathway | biological_process |
| 11. | [*TraesCS5D02G294500*](file:///\\plants.ensembl.org\triticum_aestivum\Gene\Summary%3fdb=core;g=TraesCS5D02G294500) | [GO:0003700](http://amigo.geneontology.org/amigo/term/GO:0003700) | DNA-binding transcription factor activity | molecular_function |
|  |  | [GO:0006351](http://amigo.geneontology.org/amigo/term/GO:0006351) | transcription, DNA-templated | biological_process |
|  |  | [GO:0005634](http://amigo.geneontology.org/amigo/term/GO:0005634) | Nucleus | cellular_component |
| 12. | *TraesCS5D02G308500* | [GO:0046982](http://amigo.geneontology.org/amigo/term/GO:0046982) | protein heterodimerization activity | molecular_function |
|  |  | GO:0009507 | Chloroplast | cellular_component |
|  |  | GO:0009414 | response to water deprivation | biological_process |
| 13. | [*TraesCS5D02G309500*](file:///\\plants.ensembl.org\triticum_aestivum\Gene\Summary?db=core;g=TraesCS5D02G309500) | [GO:0048046](http://amigo.geneontology.org/amigo/term/GO:0048046) | Apoplast | cellular_component |
| 14. | [*TraesCS5D02G316200*](file:///\\plants.ensembl.org\triticum_aestivum\Gene\Summary?db=core;g=TraesCS5D02G316200) | [GO:0016021](http://amigo.geneontology.org/amigo/term/GO:0016021) | integral component of membrane | cellular_component |

**Supplementary Table S6. Predicted physiochemical properties of leaf rolling proteins in wheat**

| **S.No.** | **Protein** | **Length** | **Molecular Weight (Mw)** | **Theoretical pI** | **Instability Index (II)** | **Aliphatic index (AI)** | **Grand average of hydropathicity (GRAVY)** |
| --- | --- | --- | --- | --- | --- | --- | --- |
| 1. | [*TraesCS5D02G253100*](https://plants.ensembl.org/Triticum_aestivum/Gene/Summary?db=core;g=TraesCS5D02G253100;tl=T682UKyHvLatsQmR-19507551-930821411) | 296 | 30966.64 | 7.01 | 55.62 | 50.61 | -0.534 |
| 2. | [*TraesCS5D02G052300*](https://plants.ensembl.org/Triticum_aestivum/Gene/Summary?db=core;g=TraesCS5D02G052300;tl=jhCm8c7r7Sp8rQdy-19508604-930928144) | 883 | 95733.49 | 6.73 | 47.09 | 85.89 | -0.138 |
| 3. | [*TraesCS5D02G385300*](https://plants.ensembl.org/Triticum_aestivum/Gene/Summary?db=core;g=TraesCS5D02G385300;tl=jhCm8c7r7Sp8rQdy-19508604-930928121) | 862 | 93676.55 | 6.09 | 47.94 | 84.56 | -0.178 |
| 4. | [*TraesCS5D02G320600*](https://plants.ensembl.org/Triticum_aestivum/Gene/Summary?db=core;g=TraesCS5D02G320600;tl=3jgE2PNfhcQm3lI1-19507763-930921304) | 849 | 90401.09 | 5.70 | 48.65 | 78.62 | -0.289 |
| 5. | [*TraesCS5D02G102600*](https://plants.ensembl.org/Triticum_aestivum/Gene/Summary?db=core;g=TraesCS5D02G102600;tl=zXCoeZ3UosvF3Pr6-19507644-930908380) | 1208 | 132394.27 | 8.32 | 52.19 | 78.26 | -0.143 |
